# Expression profile analysis of sheep ovary after superovulation and estrus synchronisation treatment

**DOI:** 10.1002/vms3.783

**Published:** 2022-03-19

**Authors:** Huie Wang, Xinwei Feng, Gemingguli Muhatai, Lan Wang

**Affiliations:** ^1^ College of Animal Science Tarim University Alar Xinjiang China

**Keywords:** estrus synchronisation, ovary, sheep, superovulation, transcriptome

## Abstract

Superovulation is a widely used reproductive technique in livestock production, but the mechanism of sheep's superovulation is not yet clear. Here, a method of superovulation and estrus synchronisation was used to treat female Duolang sheep. After treatment, there were significant differences in serum FSH and LH levels and the number of dominant follicles between the two groups of sheep. We identified a total of 5021 differentially expressed genes (11, 13 and 15 days after treatment) and performed RT‐qPCR analysis to identify several mRNA expression levels. GO and KEGG enrichment analysis revealed that differentially expressed genes were involved in the regulation of signalling pathways of follicular development, cell cycle, material synthesis, energy metabolism, such as *COL3A1*, *RPS8*, *ACTA2*, *RPL7 RPS6* and *TNFAIP6* may play a key role in regulating the development of follicles. Our results show a comprehensive expression profile after superovulation and estrus synchronisation treatment. We provide the basis for further research on breeding techniques to improve the ovulation rate and birth rate of livestock.

## Graphical abstract

1. Expression profile in sheep ovary after FSH treatment.

2. GO and KEGG analysis showed that some genes were associated with regulation of follicular development process.

3. TNFAIP6 May Play A Key Role In Regulating The Development Of Follicles.

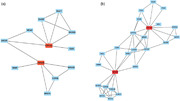

## 1 INTRODUCTION

The ovaries are female's animal reproductive organ that contains a large number of follicles at different developmental stages (Eppig, 2001; Fortune, 1994; Matzuk et al., 2002). The development of follicles presents a dynamic process of ‘follicular waves’ including recruitment, selection, dominance, turnover or ovulation (Ginther et al., 1996). This process is regulated by a variety of hormones, and the imbalance of hormone expression levels can disrupt the follicular development affecting animal reproduction (Wallach & Hodgen, 1982). Follicle stimulating hormone (FSH) is the main stimulator for mammalian follicles development (Bex and Goldman, 1975; Kauffman et al., 1998). The FSH hormone is necessary for the further development of follicles, although it is not required in the early stages of development (Norman et al., 1994). FSH mainly stimulates the growth and development of follicles, affects the number of growing follicles and stimulates the final maturation of follicles. In addition, luteinising hormone (LH) is another essential hormone that promotes oocyte maturation and ovulation (Cooke et al., 1996). FSH and LH are necessary for the cascade of gene expression necessary to initiate ovarian function (Richards & Hedin, 1988).

Ruminants, especially sheep, are widely bred as livestock in the world, providing a large number of meat, fat, milk, wool and other products for humans (Pierson & Ginther, 1988). Increasing the litter size of sheep and animal reproductive performance of sheep has always been the focus of breeding research. Many genes related to sheep reproductive capacity including Booroola gene (FecB), bone morphogenetic protein receptor type IB (BMPRIB), growth differentiation factor 9 (GDF9), bone morphogenetic protein 15 (BMP15) and Prostaglandin G/H synthase 2 (PTGS2) have been identified (Bonnet et al., 2011; Galloway et al., 2000; Hanrahan et al., 2004; Piper & Bindon, 1983; Souza et al., 2001).

Studying the mechanism of follicular development and differentiation is necessary to increase the litter size of livestock. Clinically, FSH can be used to treat infertility caused by abnormal gonadotropin secretion. In animal husbandry production, FSH can be used to perform estrus synchronisation and superovulation of livestock, which can greatly improve the litter size of livestock. In recent years, a lot of research has been conducted on sheep breeding (Li et al., 2017), but the mechanism of causing different physiological states of sheep's ovary after superovulation and estrus synchronisation treatment has not been fully understood.

Duolang sheep is an excellent dual‐purpose sheep breed for meat and fat. It has the characteristics of large size, rapid growth and development, large meat production, fresh and tender meat, early sexual maturity and high reproducibility. It is an ideal breeding breed for mutton production that is widely raised in Xinjiang, China. We used Illumina HiSeq 2500 to study Duolang sheep ovarian genes at different times after superovulation and estrus synchronisation treatment. Our research provides gene expression profiles of sheep's ovaries at various times after superovulation and estrus synchronisation treatment, which will lay the foundation for further understanding of the mechanism of sheep follicular differentiation and development.

## 2 MATERIALS AND METHODS

### 2.1 Ethical statement

#### 2.1.1 Animal and treatment

The experiment was conducted at the animal experimental station of Tarim University, and 30 Duolang ewes) 30 Duolang (2–3 years old) were used to perform this study. Ewes were randomly assigned two group either estrus synchronisation (ES, control) or super‐ovulation (SOV, treatment) (15 ewe per group). All the sheep were synchronised using Controlled Internal Drug Release Devices (CIDR) (0.3 mg, CIDR‐G® Zoetis, México; recorded as the first day) for 13 days. Prostaglandin (PG) (yofoto, Ningbo, China) was injected and CIDR was removed (Table 1). Following estrus synchronisation, the SOV group (*n* = 15) received 60 IU, 30 IU and 10 IU (200 IU total dose) of purified FSH (yofoto, Ningbo, China) in the evening/morning at 08:00 for the three consecutive days of follicular phase, while the ES group (*n* = 15) received nothing (Wang & C.S.Y.W., 2011).

**TABLE 1:** Experimental design of the two groups

	Superovulation (DC group)	Estrus synchronisation (DT group)
0 day	CIDR	CIDR
11 days	a.m. FSH injected (60 IU) p.m. FSH injected (60 IU)	
12 days	a.m. FSH injected (30 IU) p.m. FSH injected (30 IU)	
13 days	Remove CIDR PG injected (0.2 mg) a.m. FSH injected (10 IU) p.m. FSH injected (10 IU)	Remove CIDR PG injected (0.2 mg)

From the 10th to the 15th day of sheep treatment, we selected three sheep from each group (Gao et al., 2017; Ren et al., 2017; Ullah et al., 2020) to collect blood into EDTA tubes from jugular vein on an empty stomach and separate serum stored at –20°C to measure hormone content. At the same time, three ewes from each group were immediately slaughtered in the slaughterhouse, which was performed by severing the carotid artery and jugular vein, and complete ovaries were collected within 5 min. We measured and recorded diameter and number of follicles on the surface of both ovaries (Gonzalez et al., 2004), removed the surrounding fat and connective tissues, and washed the blood on the surface of the ovary with physiological saline (37°C) until the water was colourless. The ovaries were quickly frozen in liquid nitrogen and stored in a refrigerator at –80°C until RNAs extraction.

#### 2.1.2 FSH and LH hormone levels determination

The frozen serum in the refrigerator was taken out and thawed on ice. The concentrations of FSH and LH hormones were determined strictly in accordance with the protocol of the ELISA kit manufacturer (Bioss, Beijing, China) (Each sample in triplicate). Set the blank holes and sample holes to be tested during the detection. The intra‐ and inter‐assay CVs for all of the ELISA kits were less than 10%, respectively. The absorbance (OD value) at 450 nm of all samples was measured using Synergy H4 Hybrid (Biotek, Winooski, VT, USA). All samples were zeroed using blank wells and finally the corresponding concentrations were calculated according to the standard curve. The sensitivities of the FSH and LH assays were 1.0ng/ml. All data were statistically analysed using an ANOVA program SPSS 19.0.

#### 2.1.3 Library construction and sequencing

The left ovaries were rapidly ground into powder in liquid nitrogen, and total RNA was immediately extracted using TRIzol (Invitrogen, CA, USA) according to the manufacturer's protocol. The samples of ES group and SOV group were numbered DT group and DC group, respectively. The samples on the 11th, 13th and 15th day of ES group and SOV group were numbered DTA group (DT11‐1, 2 and 3), DTB group (DT13‐1, 2 and 3) and DTC group (DT15‐1, 2 and 3), and DCA group (DC11‐1, 2 and 3), DCB group (DC13‐1, 2 and 3) and DCC group (DC15‐1, 2 and 3), respectively.

RNA integrity was detected by 1% agarose gel electrophoresis and RNA Nano 6000 Assay Kit (Agilent Technologies, CA, USA) of the Bioanalyzer 2100 system. At the same time, the purity and concentration of RNA were accurately quantified using a NanoPhotometer spectrophotometer (IMPLEN, CA, USA) and a Qubit® 2.0 Flurometer (Life Technologies, CA, USA). Three micrograms of RNAs were accurately taken from each sample, and a cDNA library was prepared using NEB Next^®^ UltraTM RNA Library Preparation Kit (NEB, USA) according to the manufacturer's protocol. Library quality was assessed on an Agilent Bioanalyzer 2100 system after addition of the adaptor (Parkhomchuk et al., 2009). The post‐test library was subjected to 125 bp/150 bp paired‐end sequencing using the Illumina HiSeq 2500 (Illumina, San Diego, USA) platform.

#### 2.1.4 Data quality control and sequencing analysis

We filtered out the sequencing joints or the lower‐quality reads to obtain clean data (clean reads). At the same time, the Q20, Q30 and GC content of clean data were calculated and evaluated (Andrews, 2010). Hisat2 (v2.0.5) was used to match the filtered high‐quality cleaning data with the sheep reference genome (http://asia.ensembl.org/index.html) (Daehwan et al., 2015).

#### 2.1.5 Differential expression analysis

We used the FeatureCounts (v1.5.0‐p3) software to calculate the readings mapped to each gene by the Hisat2 software (Schmid & Grossniklaus, 2014), and then the fragments per kilobase million (FPKM) of each gene were calculated to quantify the expression of non‐genes in combination with the length information of the gene (Trapnell et al., 2012). Cuffdiff (v2.1.1) was used to calculate the FPKM of genes in each sample. The FPKM of genes were calculated by summing the FPKM of the genes in each genome. Next, differential expression in gene expression data was calculated using a negative binomial distribution model in the DESeq2 R package (1.16.1) (Love et al., 2014). Benjamini and Hochberg were used to adjust the *p* value, and genes with *p* values <0.05 were designated as differentially expressed genes (Thissen et al., 2002).

#### 2.1.6 Real‐time quantitative RCR (RT‐qPCR) analysis

Total RNAs of sheep ovaries were extracted using TRIzol (Invitrogen, CA, USA) according to the method at the time of library establishment. Required amount of RNAs was taken, and cDNA was synthesized in strict accordance with the protocol of the RT‐PCR kit (Takara, Dalian, China) manufacturer. Primers required for the experiments were designed using Primer 5 software (Table S1). We used RT‐qPCR (three copies each and three biological replicates per group) to quantify some genes (10 genes are randomly selected in each group, but only 3 differential expressions in the third group) by monitoring the product formation of the fluorescent dye SYBR Green (Takara Biotech, Dalian). β‐actin was used as an endogenous control (Passmore et al., 2009). RT‐qPCR was performed using the following reaction system: 10 μl SYBR Green (Takara, Dalian, China), 2 μl cDNA, 0.5 μl upstream and downstream primers, and 7 μl ddH_2_O water (RNase free). RT‐qPCR was performed using the following thermal cycling conditions: initially 20 s, 95°C, then 45 cycles, 95°C for 5 s, 55°C for 15 s and 72°C for 10 s. Data were then analysed using the equation 2^ΔΔCt^ (Livek & Schmittgen, 2001).

#### 2.1.7 Enrichment analysis and PPI analysis of differentially expressed genes

The ClusterProfiler R package was used to perform Gene Ontology (GO) and KEGG pathways enrichment analysis (http://www.genome.jp/kegg/) for differentially expressed genes (Xulvi‐Brunet & Li, 2009). *p* < 0.05 is considered to be significantly enriched by differentially expressed genes. At the same time, we performed PPI analysis on the differential genes based on the STRING database.

## 3 RESULTS

### 3.1 Ovarian tissue morphology

**FIGURE 1:**
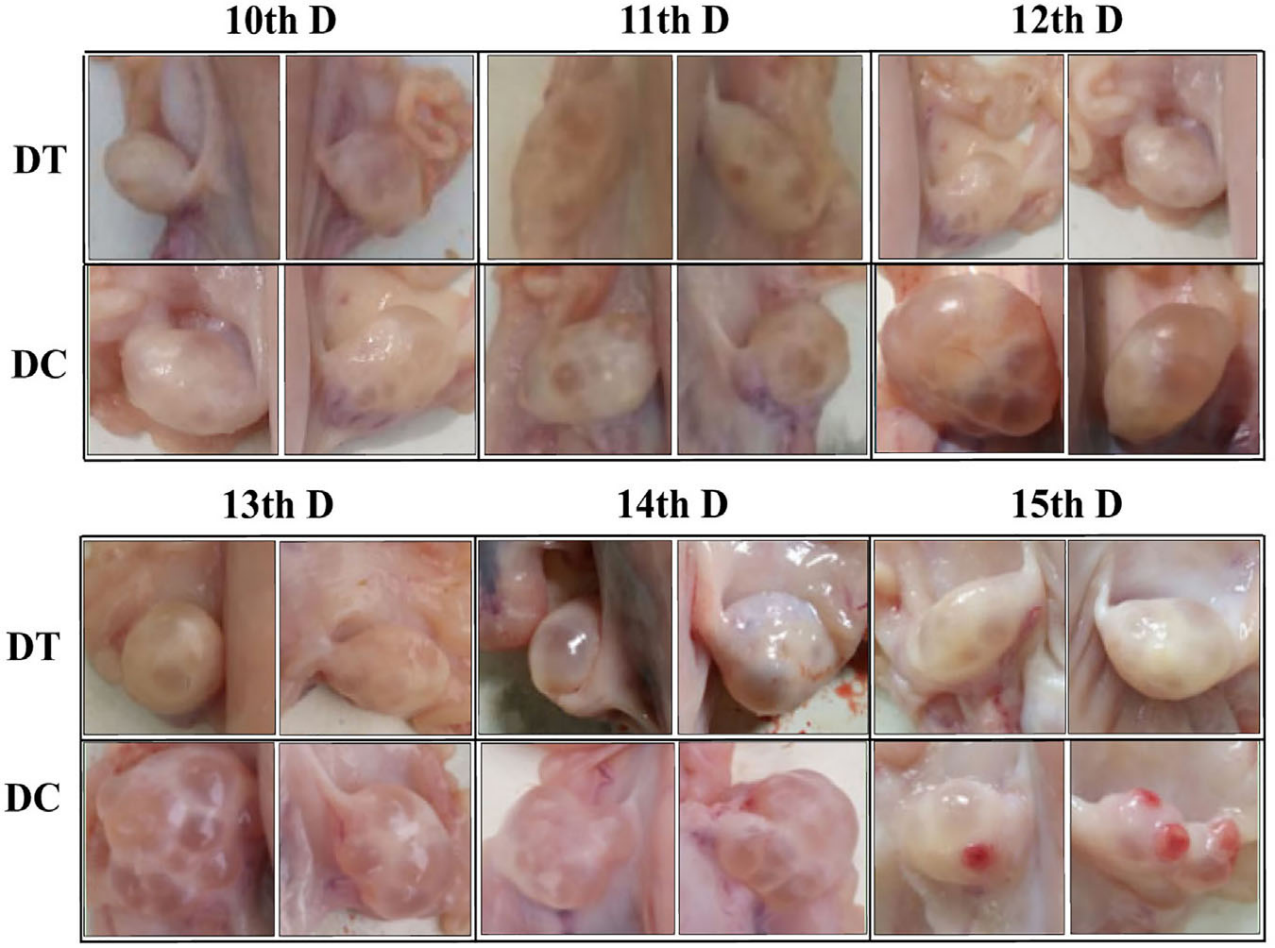
Ovarian follicular status in sheep at different time periods after estrus synchronisation and superovulation. DT is estrus synchronisation, DC is superovulation, and above the picture is the time after processing

Comparing the DC group (SOV group) and the DT group (ES group), we found that there were significant differences in the ovary morphology of the sheep between the two groups (Figure 1). The ovaries of proestrus had several follicles of different sizes (diameter <3 mm; Figure 1) on the 11th day of treatment, similar ovaries morphology in the two groups. On the 13th and 14th day after treatment, compared with the DT group, follicles on the surface of the ovary grew faster in the DC group, the wall of the follicles became thinner and the number of hemispherical follicles protruding from the surface of the ovary was more than four increased significantly (diameter >3; Figure 1). On the 15th day, we found ovaries of metoestrus had corpus hemorrhagicum and several follicles of different sizes (Figure 1) in the DC group, but in the DT group, the follicle diameter still did not reach the maximum and no ovulation was observed. These results indicate that superovulation treatment not only increases the number of follicle developments but also increases the speed of follicle growth.

### 3.2 Number of dominant follicles

To quantify the effects of both treatments on follicular development, we calculated the number of dominant follicles with a follicle diameter greater than 3 mm in both groups of sheep ovary (Gonzalez et al., 2004). We found that there were significant differences between the two groups of ovaries. By comparison, the number of dominant follicles in the DC group was significantly greater than that in the DT group from the 12th day after treatment (average increase 4.66, 15.34, 11.67 and 10 dominant follicles, respectively, *p* < 0.01) (Table 2).

**TABLE 2:** Number of dominant follicles at different time points after treatment

	11 days	12 days	13 days	14 days	15 days
DT	2.67 ± 0.58	5.67 ± 0.58	6.33 ± 0.58	4.00 ± 1.00	4.67 ± 0.58
DC	3.67 ± 1.53	10.33 ± 1.53***	21.67 ± 5.83***	15.67 ± 3.41***	14.67 ± 2.51***

*Note*: Statistical correlations were performed between DT and DC.

***p < 0.001.

### 3.3 FSH and LH hormone levels

We detected the levels of FSH and LH in the serum of sheep and found that the level of FSH in the serum of sheep in the DC group increased, while the overall level of FSH in the DT group remained unchanged. From the 13th to 15th day after treatment, the serum FSH hormone levels in the DC group were significantly higher than those in the DT group. The serum LH hormone levels in the two groups showed a tendency to increase gradually, but on the 11th–14th days after treatment, the serum LH hormone levels in the DC group increased rapidly, which was significantly higher than that in the DT group (*p* < 0.05). Interesting, on the 15th day after treatment, there were no significant difference in serum LH hormone levels between the two groups of sheep (Table 3).

**TABLE 3:** Changes in FSH and LH hormone concentrations at different time points after treatment

		0 day	10 days	11 days	12 days	13 days	14 days	15 days
FSH	DC group	69.84 ± 4.75	69.97 ± 9.35	66.54 ± 8.93	75.04 ± 6.25	82.94 ± 7.23*	80.29 ± 6.37*	80.06 ± 0.23*
	DT group	71.37 ± 6.33	67.67 ± 11.63	69.59 ± 10.79	69.62 ± 3.85	71.41 ± 3.98	71.08 ± 0.14	69.00 ± 0.53
LH	DC group	27.33 ± 21.13	12.86 ± 4.23	22.96 ± 1.42**	26.99 ± 0.59*	42.02 ± 1.41**	45.54 ± 0.67*	51.25 ± 0.54
	DT group	33.80 ± 14.93	9.72 ± 3.08	18.12 ± 1.70	24.01 ± 3.35	35.19 ± 0.69	37.45 ± 2.25	51.01 ± 1.38

*Note*: Statistical correlations were performed between DT and DC.

*p < 0.05, **p < 0.01.

### 3.4 Quality and statistics of the sequencing data

**FIGURE 2:**
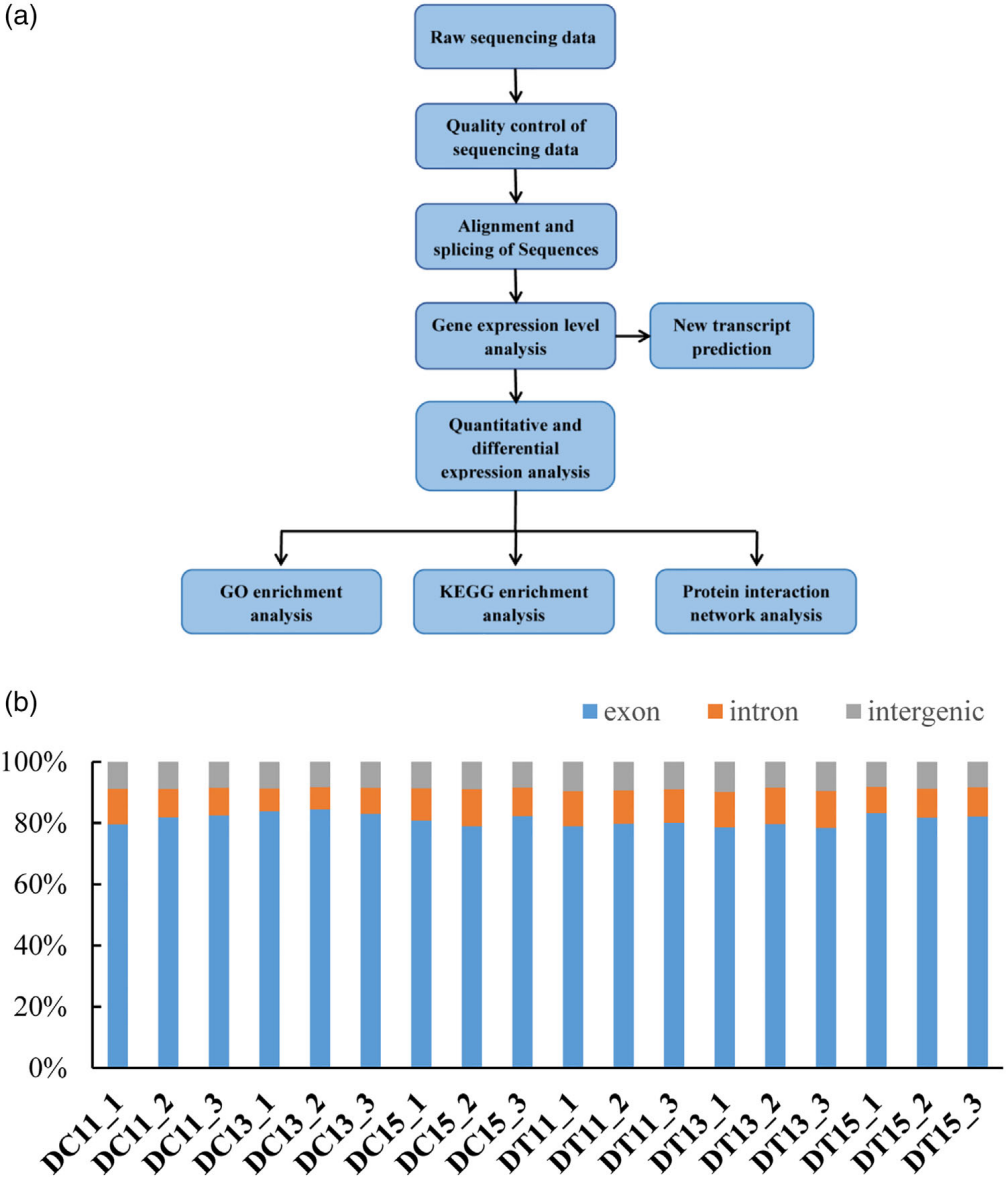
Identification of genes expression in sheep ovary treatment by RNA‐seq. (a) Flow chart of genes analysis. (b) A statistical chart of source information for all sample genes. Grey on behalf of intergenic, light red on behalf of intron and light blue on behalf of intron

According to the procedure in the pipeline, we analysed the expression patterns of genes in sheep's ovary at different time point (Figure 2a). We obtained a total of 668,709,449 raw reads and 656,489,500 clean reads (over 196G data) (Table S2). After quality control, we found that the high‐quality data (Q30) accounted for 94.46% of the total data, indicating that the quality of data produced by sequencing was good. We found that 81.12% of the RNA‐seq data was aligned with the exons, 10.09% was aligned with intron and 8.80% was aligned with intergenic sequences by mapping to the reference genome (Figure 2b). A total of 15,025 genes were identified by further analysis. Detailed information on all newly identified genes is listed in Table S3.

### 3.5 Analysis of differentially expressed genes

**FIGURE 3:**
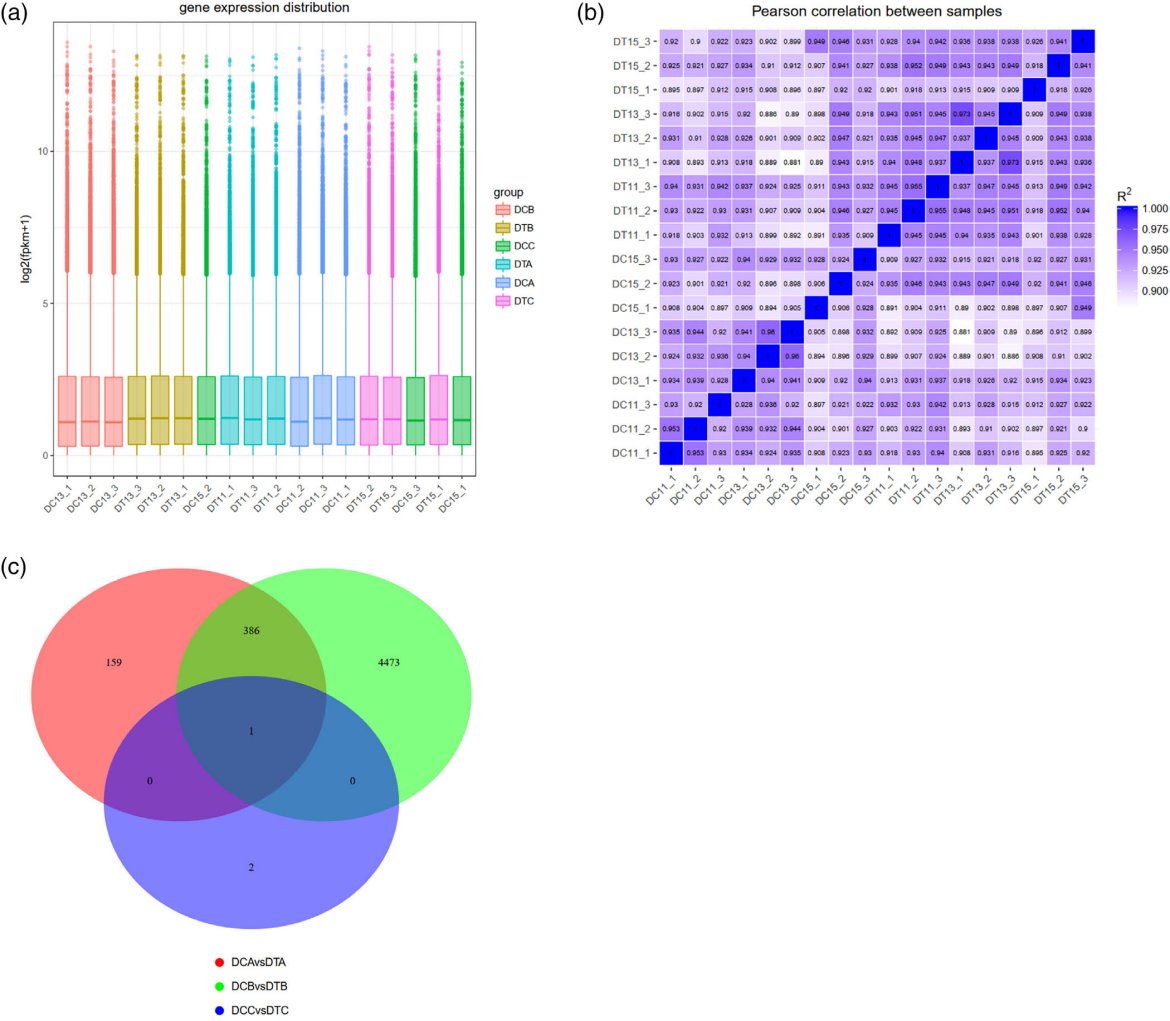
The information of genes in sheep at different time points after estrus synchronisation and superovulation. (a) Under different experimental conditions, the horizontal box plot is expressed, where the abscissa is the sample (group) name, the ordinate is log2 (FPKM+1), and the box plot of each region is for the five statistics (top to bottom: value, upper quartile, median, lower quartile and minimum, respectively). (b) Sample correlation heat map. (c) Differential gene Venn diagram. The sum of all the numbers in the circle represents the total number of differential genes in the comparison combination, and the overlapping regions represent the differential genes shared between the combinations. Red representative DCA vs. DTA, green representative DCB vs. DTB and blue representative DCC vs. DTC (*p* < 0.05)

The gene expression patterns of the samples at the overall level of the different sample genes were shown in Figure 3a. Correlation analysis shown that the minimum *R* ^2^ of these samples were 0.881 (>0.8) (Figure 3b) and indicated that the samples we selected had good biological reproducibility (Pearson, 1897). Compared with the DT group, 546 (144 upregulated and 402 downregulated), 4860 (2019 upregulated and 2841 downregulated) and 3 (1 upregulated and 2 downregulated) differentially expressed genes were identified in sheep ovaries on 11th, 13th and 15th day after treatment (*p* < 0.05), respectively (Figure S1). And there was only one gene (TNFAIP6) that differed in the ovaries of these three periods (Figure 3c). All identified differentially expressed genes are listed in Table S4.

### 3.6 Verify differential expression genes

**FIGURE 4:**
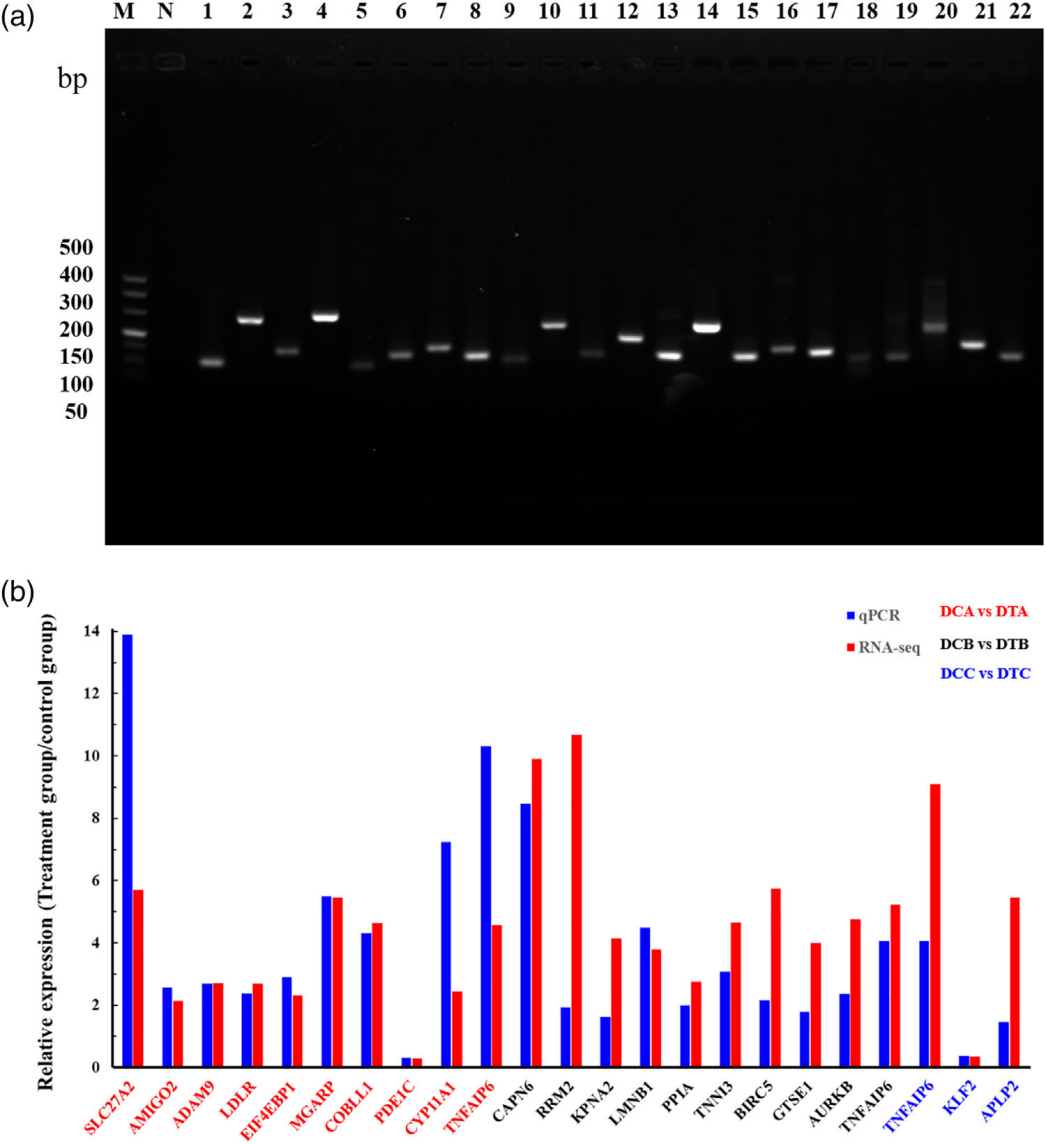
Validation of genes and their differential expression in sheep ovary. (a) RT‐PCR amplification of genes with specific primes. The order of electrophoresis are Marker (Takara DL500 500 bp, 400 bp, 300 bp, 200 bp, 150 bp, 100 bp and 50 bp), N is negative control, lane 1 is β‐actin, lanes 2–22 represent *SLC27A2*, *AMIGO2*, *ADAM9*, *LDLR*, *EIF4EBP1*, *MGARP*, *COBLL1*, *PDE1C*, *CYP11A1*, *CAPN6*, *RRM2*, *KPNA2*, *LMNB1*, *PPIA*, *TNNI3*, *BIRC5*, *GTSE1*, *AURKB*, *TNFAIP6*, *TNFAIP6*, *KLF2* and *APLP2*, respectively. (b) Expression of 23 differentially expressed genes by RT‐qPCR. Blue is the result of qPCR, and red is the result of RNA‐seq (*p* < 0.05). The red gene name represents the DCA vs. DTA group; the black gene name represents the DCB vs. DTB group and the blue gene name represents the DCB vs. DTB group. The ordinate is the ratio of the relative expression levels of the gene in the treatment group and the estrus synchronisation (DTN/DCN, N stands for a, b and c)

To determine the accuracy of the RNA‐seq results, we confirmed the expression levels of these genes by further experiments. We randomly selected 10 differentially expressed genes from each sample to verify (except DCC vs DTC, only 3 differentially expressed genes). By gel electrophoresis analysis, the reverse transcription PCR (RT‐PCR) products showed a single band of the expected size, which indicated that the primers for these genes are accurate and specific (Figure 4a). We further validated these amplification products using DNA sequencing. Compared with RNA‐seq data, the results of qPCR suggested that all 23 genes amplified showed the expected difference (Figure 4b). There is a strong agreement between the results of these experiments and the RNA‐seq data, indicating that the RNA‐seq data can truly reflect the true differential expression in sheep.

### 3.7 Functional enrichment analysis of differentially expressed genes

**FIGURE 5:**
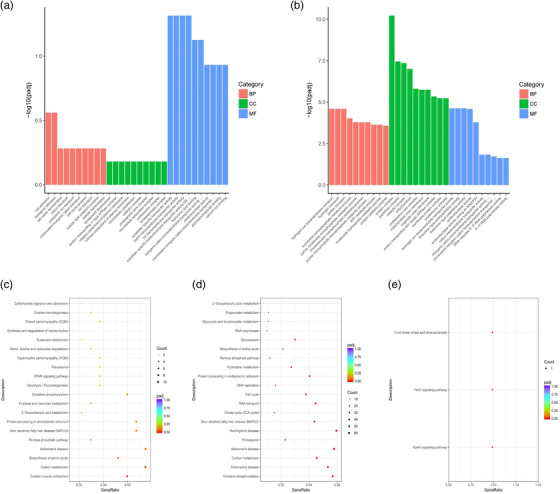
Enrichment analysis of differentially expressed genes sheep at different time periods after estrus synchronisation and superovulation. GO enrichment analysis of differentially expressed genes at DCA vs. DTA (a) and DCB vs. DTB (b) groups (*p* < 0.05). The abscissa is the GO macromolecules and GO term at the next level, and the ordinate is the number and proportion of genes annotated to the term. KEGG enrichment analysis was performed on the hosting genes of differentially expressed genes in DCA vs. DTA (c), DCB vs. DTB (d) and DCC vs. DTC (e) groups. The vertical axis and the horizontal axis represent the name of the access and the access factor, respectively. The size of the dots represents the number of genes that are enriched in the access, and the colours correspond to different *q*‐values

**FIGURE 6:**
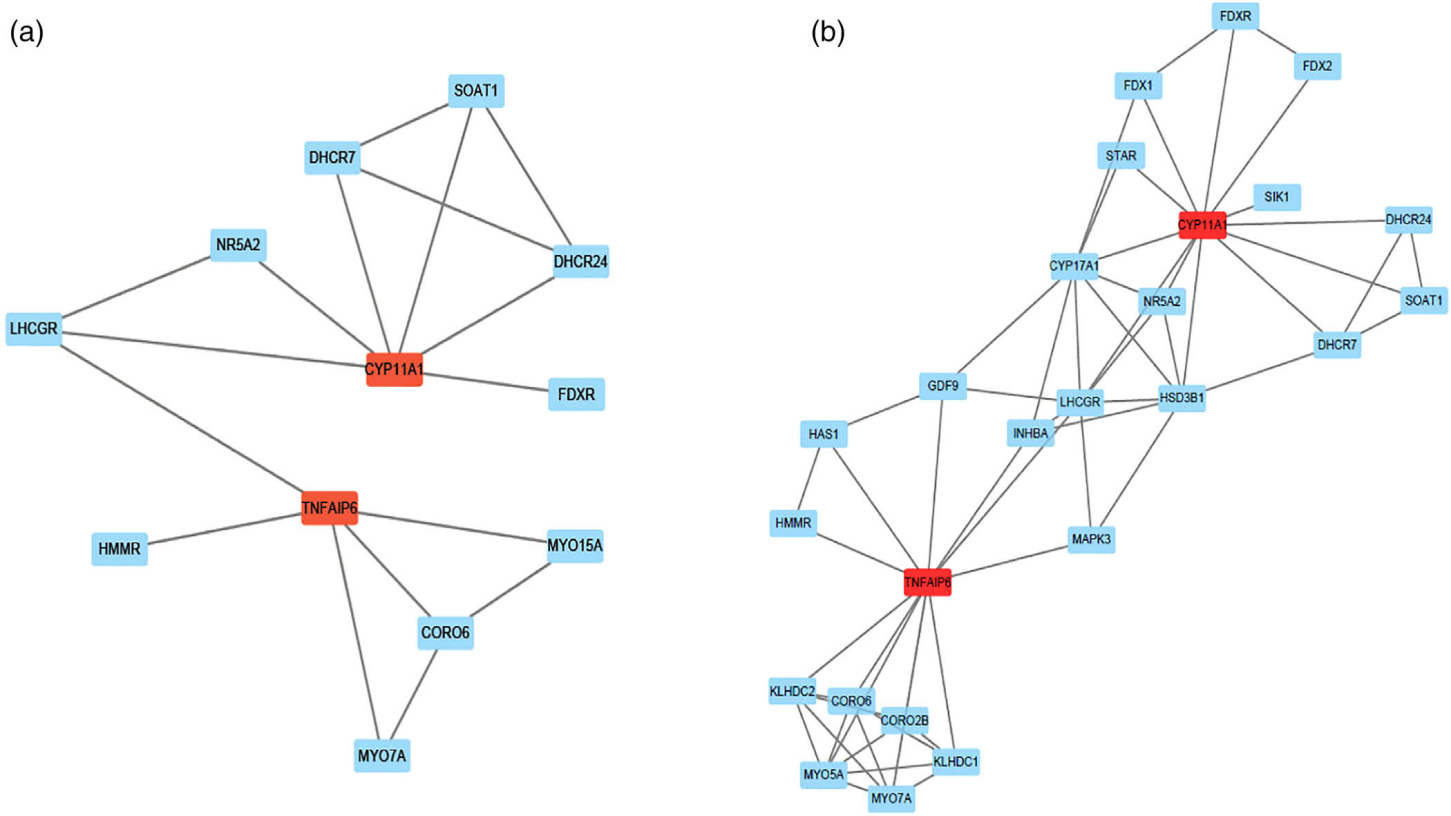
**Partial network diagram of PPI analysis**. (a) Partial network diagram of DCA vs. DTA differentially expressed genes. (b) Partial network diagram of DCB vs. DTB differentially expressed genes. The red squares represent TNFAIP6 and CYP11A1

To further understand the biological functions of these differentially expressed genes in the ovary between DC and DT group, we used the GO and KEGG pathways to enrich these differentially expressed genes. The results of GO annotation showed 46 (DCA vs DCT group) and 240 (DCB vs DTB group) significantly different genes in biological process, molecular function and cellular component (Table S5) (*p* < 0.05). The differential genes in the DCA vs DTA group were mostly enriched in molecular function (endopeptidase complex, substrate‐specific transporter activity, cation transmembrane transporter activity and ion transmembrane transporter activity), while DCB vs DTB, the most enriched in the group, were the cellular composition (protein complex, peptidase complex and catalytic complex) (Figure 5a and b). Especially in the DCB vs DTB group, the biological process of sheep ovary was the most active during this period. KEGG enrichment analysis was used to analyse the biological functions involved in these differential genes (Table S6). At different stages after treatment, differentially expressed genes were significantly enriched in signalling pathways involved in follicular development and maturation, such as the FoxO signalling pathway, Apelin signalling pathway, and ovarian steroidogenesis (Table 4). These results suggest that sheep ovary after FSH treatment has a strong biological response (Figure 5C–E). We found a large number of protein–protein interactions on 11th and 13th days after treatment by PPI analysis of differentially expressed genes in sheep ovaries at different stages of treatment, some of the main genes were shown in Figure 6 and all the interaction networks were shown in Figure S2 (Table S7).

**TABLE 4:** Differentially expressed genes are significantly enriched in pathways primarily related to follicular development and maturation

Pathways	Enriched differential genes		
	DCA vs. DTA	DCB vs. DTB	DCC vs. DTC
**Pelin signalling pathway**	MYLK4	MYLK4, ACTA2, PRKAG3, GNAI3, GNB4, GNG4, GNAI1, LOC105614854, SMAD3, LOC101108001, TGFBR1, PLCB3, MTOR, PLAT, GABARAPL2, GNG5, GABARAP, RPS6, SLC8A2, RYR2	KLF2
**Ovarian steroidogenesis**	CYP11A1, LDLR, PLA2G4B, PLA2G4D	HSD3B1, PLA2G4B, LDLR, PLA2G4A, CYP17A1, CYP2J, CYP1A1, HSD17B7, CYP11A1,GDF9, LOC101121563	
**FoxO signalling pathway**	CDKN2D, FBXO32, GADD45G, FOXO4, PIK3R1	PLK4, ATG12, CDKN2D, PCK2, CCNB3BCL2L11, LOC101102473, GADD45G, PRKAG3, FOXO4, IRS2, GADD45A, TGFBR2, PIK3CB, SMAD3, PIK3R3, CAT, BCL6, TGFBR1, CDK2, CHUK, LOC443240, GABARAPL2, GABARAP, SKP2	KLF2
**Carbon metabolism**	GPT, TKTL1, HK2, FBP1, ENO2, ALDOB, ME1, PSAT1, GOT1, ECHS1	MDH2, GOT2, ALDOA, ME1, PRPS1, CS, SUCLG2, PGP, ECHS1, SDHC, DLAT, LOC101114294, SUCLG1, DLD, ACO2, PSAT1, GOT1 GCSH, PCCB, LOC101110145, ENO1, GPI, ACADS, MDH1, OGDH, PFKP, PFKL, ALDOB, PSPH, HADHA, ENO3, TKT, RGN, IDH3B, MUT, HK2, PDHA1, G6PD, SUCLA2, IDH2, TKTL1, CAT, MCEE, ACAT1, PHGDH, GPT, FH, OGDHL, FBP1, RPE, GLUD1, IDH3G, ALDH6A1	

## 4 DISCUSSION

In recent years, with the rapid breakthrough and development of high‐throughput technology, many studies have revealed important issues in many livestock production processes by RNA‐seq (Clark et al., 2017; Jäger et al., 2011; Fan et al., 2013). Increasing the reproductive rate of livestock can bring huge economic benefits (Nienaber & Hahn, 2007), estrus synchronisation and superovulation are widely used in livestock production to increase the reproductive capacity of livestock (Mendoza‐Tesarik & Tesarik, 2018). In this study, the follicle development rate and the number of ovulations between the two groups were significantly different. This is consistent with the known function of FSH, but it was possible to promote follicular development and maturation, and we used RNA‐seq to further study its molecular level regulation.

After FSH treatment, we found that the number of differentially expressed genes first increased and then decreased. There were 546 differentially expressed genes between the two groups immediately after FSH treatment (DCA vs. DTA). However, KEGG pathway enrichment analysis showed that only Cardiac muscle contraction, Carbon metabolism and Biosynthesis of amino acids were significantly enriched. Although some differential expressed genes were enriched in PPAR signalling pathway, synthesis and degradation of ketone bodies, ovarian steroidogenesis and cell cycle, these differences had not reached a significant degree. Biosynthesis of amino acids is an important part of protein synthesis. These results indicated that the protein biosynthesis of the ovary is enhanced after FSH treatment, which is consistent with the previous report that FSH can accelerate protein biosynthesis within 1 h (Means & Hall, 1967). FSH may promote ovarian protein biosynthesis and may be an important way to regulate follicular development.

The number of differentially expressed genes in the two groups of ovaries was then increased after receiving more FSH treatment (DCB vs. DTB). We found that these genes involved in follicular development and premature activation were significantly upregulated, including COL3A1, RPS8 and ACTA2, RPL7 and RPS6, indicating high concentrations of FSH promote the development of follicles by stimulating their early entry into the developmental stage, which is consistent with previous reports (Diao et al., 2004; Mork et al., 2012; Reddy et al., 2008; Terenina et al., 2016; Yoon et al., 2006). KEGG enrichment analysis showed that differentially expressed genes such as CYP11A1, LDLR, SMAD3, GDF9, ACTA2, RPS6 and CDK2 were significantly enriched in the pelin signalling pathway, and ovarian steroid production, FoxO signalling pathway and carbon metabolism were directly related to follicular development and maturation (Dupont et al., 2013; Kwong et al., 2010; Robinson & Etches, 1986; Sim & Denlinge, 2008). CYP11A1 plays a key role in regulating ovarian steroid production, and in the mitochondria, CYP11A1 can convert cholesterol into pregnenolone to regulate progesterone synthesis (Assidi et al., 2008; Manna et al., 2016). Previous studies have shown that CYP11A1 will lead to an increase in aromatase expression and serum estradiol levels in female mice, which will lead to an increase in the number of mature follicles. At the same time, the expression level of CYP11A1 in the follicles of multiple goats is higher than that of uniparous goats (Bakhshalizadeh et al., 2018; Zou et al., 2020). This may be the reason why the expression level of CYP11A1 in sheep ovary increased significantly after treatment with FSH. In addition, studies have reported that GDF9 is hardly expressed when oocytes develop into primordial follicles. And the oocytes expressing GDF9 in the later development are obviously larger. GDF9 sends signals through SMAD 2 and 3 to regulate oocyte development (Coutts et al., 2008). This is consistent with our results that the expression levels of GDF9 and SMAD3 were not different between the two groups immediately (DCA vs DTA) after FSH treatment, but they increased significantly with the increase of FSH treatment (DCB vs DTB). At the same time, these differentially expressed genes extensively enrich in signalling pathways related to metabolism, such as energy metabolism, cell cycle, cell proliferation and biomass synthesis, such as phosphorylation, carbon metabolism, RNA transport, cell cycle and DNA replication. These results indicated that FSH regulates the development of follicles through a wide range of regulatory pathways.

But, there was a significant difference in the expression of only three genes (APLP2, TNFAIP6 and KLF2) between the two groups as ovulation occurred (DCC vs. DTC). This indicated the expression level of differentially expressed genes caused by FSH treatment decreased. The occurrence of ovulation after removal of FSH treatment led to the rapid recovery of differentially expressed genes between the two groups, indicating that FSH plays a key role in superovulation. This also verifies that the above‐mentioned difference between DTB and DTC is mainly caused by FSH treatment, which further indicates that FSH treatment can promote the maturation of follicles by regulating cell proliferation, cell cycle and energy metabolism.

Interestingly, we found that TNFAIP6 was the only gene that was upregulated at all three treatment time points. Previous reports have shown that the expression of TNFAIP6 in follicles of multiple goats during estrus is eight times higher than uniparous goats (Zou et al., 2020), and the lack of TNFAIP6 will cause mice to be sterile (Yu et al., 2010). These studies have shown that TNFAIP6 is deeply related to the ovulation number and fertility of female animals. LH treatment inhibits the expression of FSHr and Lhcgr and weakens the effect of FSH; even in the early stage of follicular development, it may cause follicular atresia and cysts (Farhat et al., 2011). We believe that TNFAIP6 plays a key role in superovulation, although this requires further experimental verification. Simultaneously, CYP11A1 and TNFAIP6 are the core of the interaction network and interact with many important genes that regulate follicle development. Once again, they show the key role in these candidate genes. The specific mechanism by which these genes regulate superovulation in sheep will be the focus of our next research.

In conclusion, our ovarian transcriptome studies of the ovary at different time points after superovulation and estrus synchronisation treatment indicate that TNFAIP6 and CYP11A1 may play an important role in promoting development of sheep follicle. At the same time, LDLR, SMAD3, GDF9, ACTA2, RPS6 and CDK2 also play an active role in this physiological activity. According to GO and KEGG analysis, FSH can also promote the development of follicles through a combination of signalling pathways that regulate cell proliferation, cell cycle and energy metabolism. Our research provides valuable resources for further understanding of the mechanism of superovulation during sheep follicular maturation.

## CONFLICT OF INTERESTS

The authors have declared that no competing interests exist.

## ETHICAL STATEMENT

All experiments were performed according to the regulations for the Administration of Affairs Concerning Experimental Animals (Ministry of Science and Technology, China; revised in August 2011) and approved by the Institutional Animal Care and Use Committee of Tarim University, Alar, China.

## AUTHOR CONTRIBUTIONS

Huie Wang: conceptualisation; data curation; formal analysis; funding acquisition; writing – original draft; writing – review & editing. Gemingguli Muhatai: investigation; methodology. Lan Wang: methodology; validation; visualisation.

### PEER REVIEW

The peer review history for this article is available at https://publons.com/publon/10.1002/vms3.783.

## Supporting information

SUPPORTING INFORMATION

SUPPORTING INFORMATION

SUPPORTING INFORMATION

## Data Availability

The data sets generated during and/or analysed during the current study are available from the corresponding author on reasonable request.
