# Supplementary figures and images for: Expression profile analysis of sheep ovary after superovulation and estrus synchronisation treatment
Source: Vet Med Sci. 2022 Mar 19;8(3):1276–87. doi: 10.1002/vms3.783 (PMC9122410; doi:10.1002/vms3.783)

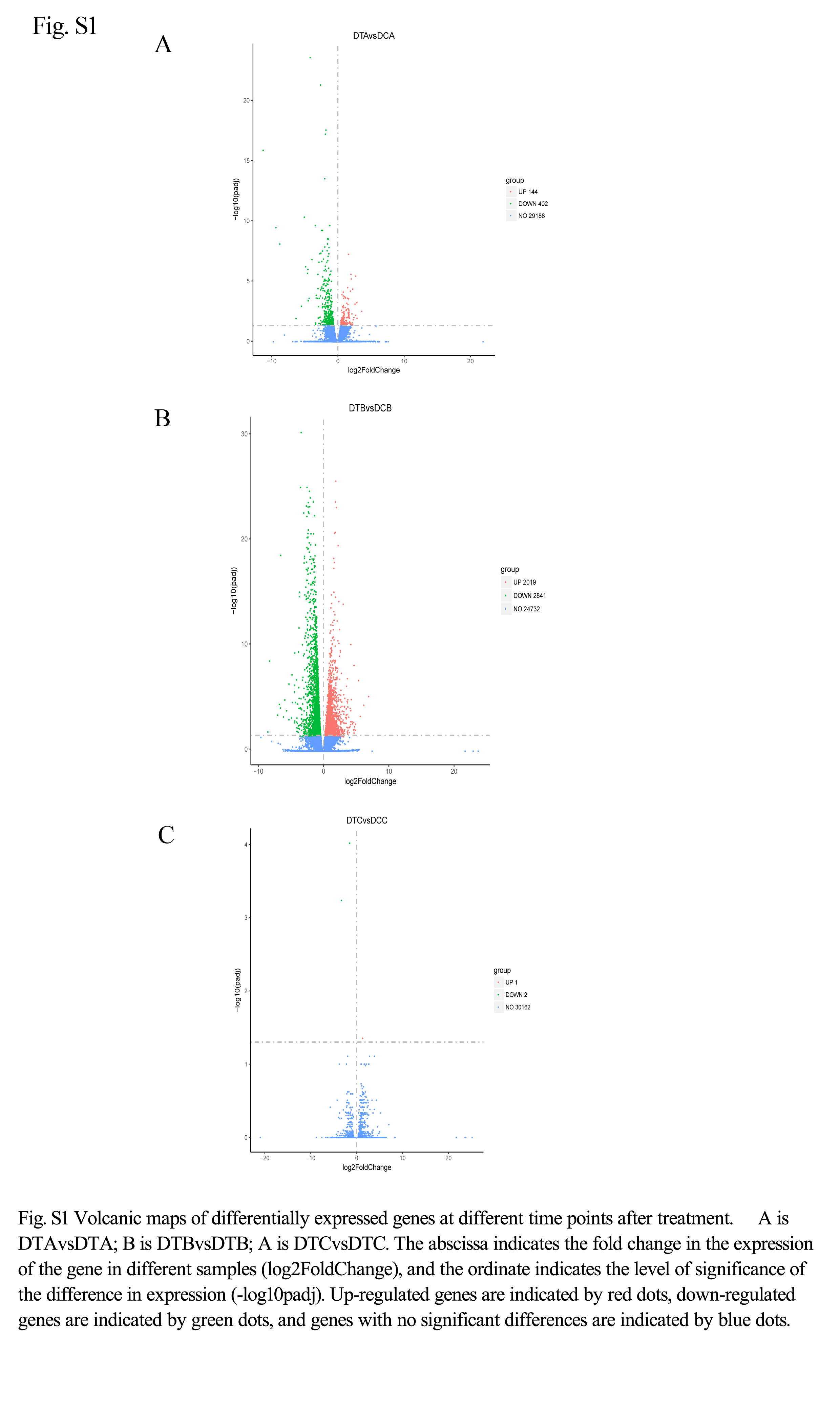

Supplement: Supplementary file 1 — SUPPORTING INFORMATION [file VMS3-8-1276-s003.tif]

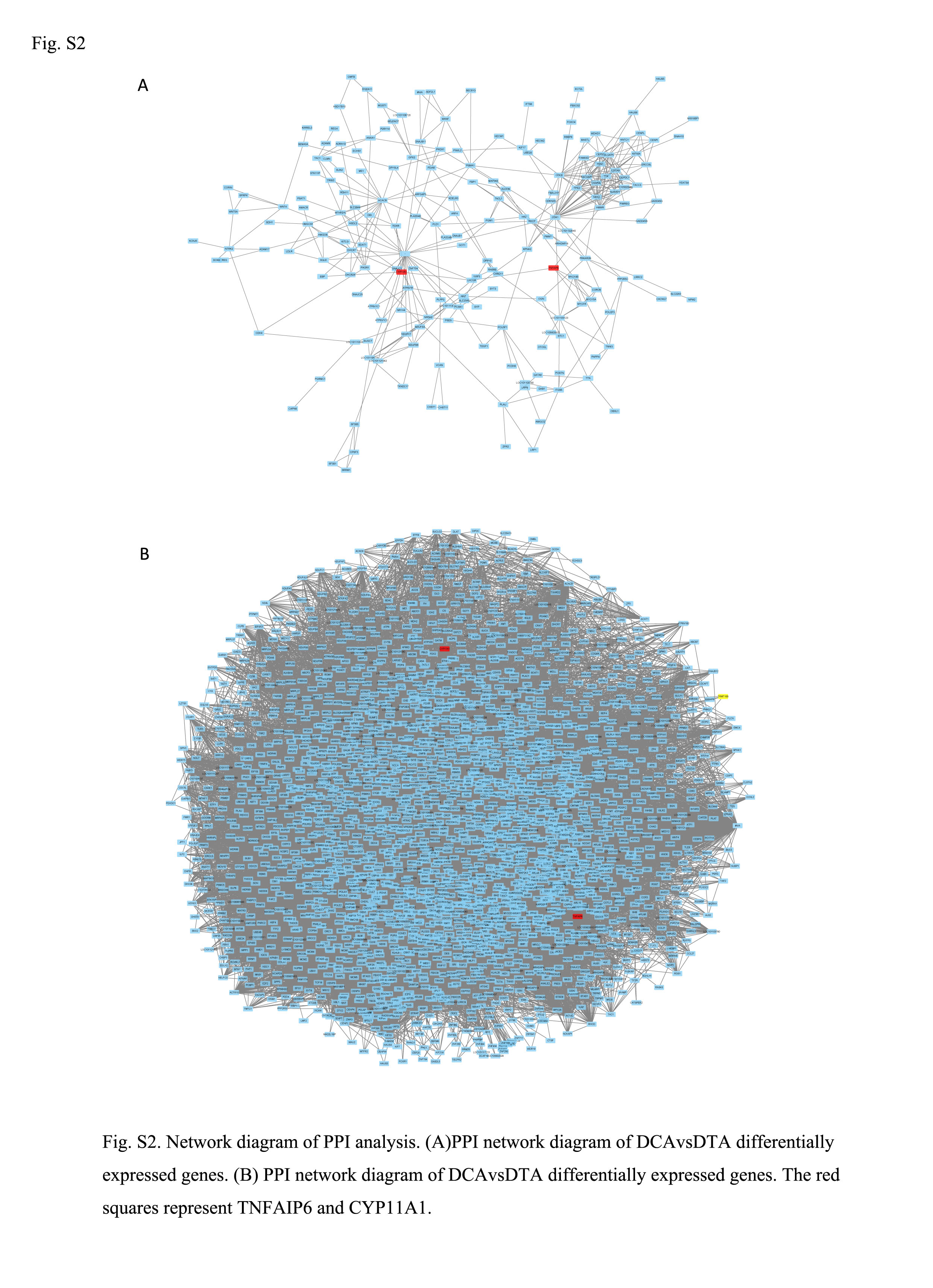

Supplement: Supplementary file 2 — SUPPORTING INFORMATION [file VMS3-8-1276-s001.jpg]
